# Chest radiography practice in critically ill patients: a postal survey in the Netherlands

**DOI:** 10.1186/1471-2342-6-8

**Published:** 2006-07-18

**Authors:** Marleen E Graat, Karin A Hendrikse, Peter E Spronk, Johanna C Korevaar, Jaap Stoker, Marcus J Schultz

**Affiliations:** 1 Department of Intensive Care Medicine; Academic Medical Center, University of Amsterdam. Amsterdam, The Netherlands; 2 Department of Radiology; Gelre Hospitals, Location Lukas, Apeldoorn, The Netherlands; 3 Department of Intensive Care Medicine; Gelre Hospitals, Location Lukas, Apeldoorn, The Netherlands; 4 HERMES Critical Care Group, Amsterdam, The Netherlands; 5 Department of Clinical Epidemiology and Biostatistics; Academic Medical Center, University of Amsterdam. Amsterdam, The Netherlands; 6 Department of Radiology; Academic Medical Center, University of Amsterdam. Amsterdam, The Netherlands; 7 Laboratory of Experimental Intensive care and Anesthesiology; Academic Medical Center, University of Amsterdam. Amsterdam, The Netherlands

## Abstract

**Background:**

To ascertain current chest radiography practice in intensive care units (ICUs) in the Netherlands.

**Methods:**

Postal survey: a questionnaire was sent to all ICUs with > 5 beds suitable for mechanical ventilation; pediatric ICUs were excluded. When an ICU performed daily-routine chest radiographs in any group of patients it was considered to be a "daily-routine chest radiography" ICU.

**Results:**

From the number of ICUs responding, 63% practice a daily-routine strategy, in which chest radiographs are obtained on a daily basis without any specific reason. A daily-routine chest radiography strategy is practiced less frequently in university-affiliated ICUs (50%) as compared to other ICUs (68%), as well as in larger ICUs (> 20 beds, 50%) as compared to smaller ICUs (< 20 beds, 65%) (*P *> 0.05). Remarkably, physicians that practice a daily-routine strategy consider daily-routine radiographs helpful in guiding daily practice in less than 30% of all performed radiographs. Chest radiographs are considered essential for verification of the position of invasive devices (81%) and for diagnosing pneumothorax, pneumonia or acute respiratory distress syndrome (82%, 74% and 69%, respectively). On demand chest radiographs are obtained after introduction of thoracic drains, central venous lines and endotracheal tubes in 98%, 84% and 75% of responding ICUs, respectively. Chest films are also obtained in case of ventilatory deterioration (49% of responding ICUs), and after cardiopulmonary resuscitation (59%), tracheotomy (58%) and mini-tracheotomy (23%).

**Conclusion:**

There is notable lack of consensus on chest radiography practice in the Netherlands. This survey suggests that a large number of intensivists may doubt the value of daily-routine chest radiography, but still practice a daily-routine strategy.

## Background

Chest radiographs are frequently obtained in intensive care unit (ICU)-patients [1]. Two different schools of thought exist regarding the utility of chest radiographs in these patients: chest radiographs should be obtained on indication only, specifically when there is a sound reason to obtain a film (so-called "on demand" chest radiographs) – or chest radiographs should be obtained routinely every day, that is without any specific reason (so-called "daily-routine chest radiographs"). Argument for the latter strategy is the high prevalence of findings on chest radiographs of ICU-patients [2]. However, although chest radiographs have high diagnostic accuracy for detecting malposition of indwelling devices like translaryngeal tubes or central venous catheters [3], diagnostic accuracy in regard to other abnormalities such as cardiogenic edema, pneumothorax and pleural effusion is low [4]. Radiographic abnormalities on chest films are dependent on lung inflation at the time the chest radiograph is obtained. Indeed, higher positive end-expiratory pressure levels may erroneously give the impression that acute respiratory distress syndrome (ARDS), infective infiltrates, or permeability edema are resolving, when radiographic translucency has increased [5]. In addition, we recently demonstrated that daily-routine chest radiographs hardly ever reveal unexpected clinical important abnormalities and seldom result in a change in therapy [4].

We suspected major differences in chest radiography practice between ICUs in the Netherlands, both in regard to practice of chest radiographs as well as thoughts on diagnostic yield. Therefore, we performed a postal survey among Dutch non-pediatric ICUs.

## Methods

In January 2005, an anonymous simple questionnaire was sent to the medical directors of all non-pediatric ICUs with > 5 beds suitable for mechanical ventilation in the Netherlands. Addresses were retrieved from the Dutch Intensive Care Society. A total of 65 ICUs received the questionnaire. After two months, a postal reminder was sent, including a copy of the survey. The anonymity of this survey precluded analysis of non-responders.

The questionnaire comprised demographic questions regarding the hospital and ICU-setting, and questions on chest radiographs practice [see Additional file 1]. A "daily-routine chest radiography strategy" was defined as the performance of chest radiographs without a specific reason, i.e., standing orders for obtaining daily chest radiographs in all patients or certain patient groups (medical patients, general surgery patients, neurosurgery patients, or patients after cardiac surgery). An "on demand chest radiography strategy" was defined as the performance of chest radiographs only on clinical indication such as placement of invasive instruments or a change in oxygenation status. Per definition, when in an ICU daily-routine chest radiographs were obtained, this unit was regarded to practice a daily-routine chest radiography, irrespective of whether this strategy was performed in all patients or only certain patient groups.

Analysis was performed on anonymous data with SPSS version 11.5.1. Categorical and continuous variables were compared using χ^2 ^Fisher's Exact, and Student's t test, where appropriate. A *P*-value of less than 0.05 was considered to represent statistical significance.

## Results

### ICU characteristics

Of the responding ICUs (N = 43, 66%), 2 turned out to have < 5 beds suitable for mechanical ventilation at the moment of the survey (these were excluded, leaving 41 ICUs for final analysis); 34 had 5 – 15 beds, 3 had 16 – 20 beds, and 4 had > 20 beds available for mechanical ventilation. Ten ICUs were located in academic hospitals (ICUs in university-affiliated hospitals) (response of 100%); 31 hospitals were non-academic (response 56%). Forty ICUs were so-called "closed format" departments (i.e., the patients were under the direct care of a team of intensivists). In 7 ICUs fellows in ICU-medicine were trained.

### On demand versus daily-routine chest radiograph strategy

A daily-routine chest radiograph strategy was practiced in 50% of the academic ICUs and in 68% of non-academic ICUs (*P *> 0.05) (Table 1). Larger ICUs practiced an on demand strategy more often than smaller ICUs (*P *> 0.05). In 21 out of 26 ICUs that practiced a daily-routine strategy, chest radiographs were obtained in all patients or only those that were intubated and mechanically ventilated; in 5 ICUs it was policy to perform a daily-routine strategy in only distinct patient groups (one center specified this group as those after cardiopulmonary surgery).

### Abnormalities for which chest radiographs are judged to be essential

In the majority of responding ICUs chest radiographs were considered to be essential for verification of the position of central venous catheters and chest tubes (77% and 81%, respectively). In 84%, 72% and 69% of responding ICUs chest radiograph were judged essential for diagnosing pneumothorax, pneumonia, or ARDS, respectively. There were no statistical significant differences among ICU-types.

### Indications for on demand chest radiographs

On demand CXRs were obtained after insertion of invasive devices, such as chest tubes, central venous lines and endotracheal tubes in 98%, 84% and 75% of the responding ICUs, respectively. In 49% of the responding ICUs, these chest radiographs were obtained after worsening of oxygenation, in 59% after cardiopulmonary resuscitation, in 58% after tracheotomy and in 23% after mini-tracheotomy. No statistical significant differences were found amongst ICU-types, except for chest radiographs after insertion of central venous catheters, which were obtained more frequently in non-academic ICUs (*P *< 0.05).

### Assumed value of daily-routine chest radiographs

In general, the value of daily-routine chest radiographs was suggested to be low. In approximately half (49%) of the responding ICUs it was believed that not more than 30% of these films truly influence daily management (Table 2). The difference in this opinion between academic and non-academic ICUs was not statistically significant. Opinion on the value of daily-routine chest films was not influenced by the volume of chest radiographs (i.e., the number of beds per ICU), or the presence or absence of ICU-fellows. Although in ICUs with daily interdisciplinary meetings, daily-routine chest radiographs were considered to be more valuable than in ICUs that lack such meetings, difference were not statistically significant.

### Assumed value of on demand chest radiographs

The value of on demand chest radiographs was also suggested to be low (Table 2). In 70% of the responding ICUs it was believed that less than 30% of on demand chest radiographs lead to a change in therapy. There was no difference in opinion on the value of on demand chest radiograph between academic and non-academic hospitals, nor was this influenced by volume or the presence or absence of ICU-fellows.

## Discussion

This survey suggests that the practice of and opinion on (daily-routine) chest radiographs varies remarkably among ICUs in the Netherlands. More than half of the responding ICUs practiced some sort of daily-routine strategy, though not in all patients. In most ICUs the value of daily-routine chest radiograph is doubted.

Although only two thirds of the questionnaires were returned, the distribution of different hospitals and ICUs was comparable to the total group. The ICUs that did return the survey, however, included all of the academic ICUs and most of the ICUs that train ICU-fellows. Because the survey was held anonymously, it was only possible to send a general reminder to the whole group of ICUs and not a specific one to the ICUs that had not already returned the survey. Importantly, this study reflects the opinion of ICU-directors, and is therefore possible that the results of the survey do not reflect the opinions of all categories of intensivists. However, all directors were clinical directors, and moreover, all directors were registered intensivists. Finally, this study only contains declarative data and no objective data.

In view of the fact that in the majority of hospitals daily-routine chest radiographs are obtained, it is remarkable to see that more than half of the respondents consider the influence of these chest radiographs on the clinical management to be low. This opinion is in contrast with several reports on the value of daily-routine chest radiographs [6-20], but very well in line with a recent study of our group [4]. Indeed, the reported efficacy of daily-routine chest radiographs varies remarkably; on average the incidence ratio of total findings is approximately 35% [2]. Not all findings, however, are of clinical importance: findings may be old (i.e., already present on previous films) or reflect only minor (patho)-physiological changes. In a recent study in a mixed medical-surgical ICU, we showed that only 5.8% of daily-routine chest radiographs revealed a new and clinically important abnormality [4]. More importantly, in this study only 2.2% of daily-routine chest radiographs had a major abnormality that truly influenced patient management.

The consensus opinion of the American College of Radiology-expert panel is that daily-routine radiographs are indicated for patients with acute cardiopulmonary problems and for patients receiving mechanical ventilation [21] (update in progress). In stable patients admitted for cardiac monitoring, or in stable patients admitted for extra-thoracic disease only, an initial admission radiograph is recommended, with follow-up radiographs obtained only for specific cardiopulmonary indications. At present there are no international critical care society guidelines regarding utilization of (daily-routine) chest radiographs in critically ill patients. Two studies compared a daily-routine strategy directly with a restrictive strategy (in which chest radiographs were taken only if clinically indicated) [20,22]. In a prospective, randomized, observational study, Krivopal *et al *determined whether there was any difference in diagnostic, therapeutic, and outcome efficacy between a daily-routine and a non-routine chest radiography strategy in mechanically ventilated medical patients [20]. Patients were randomly assigned to have their chest radiographs performed routinely every morning and on clinical indication, or only on clinical indication and after insertion of invasive devices. There was no difference in the mean duration of mechanical ventilation, and length of stay in ICU between the two groups. In a prospective, non-randomized, controlled study in a pediatric intensive care unit, Price *et al *determined the impact of change in chest radiograph ordering. Ordering changed from a daily-routine chest radiography policy to no standing orders for routine daily morning chest radiographs (i.e., each radiograph required a written order and a clinical indication) [22]. They found no significant changes in average length of stay in ICU, nor in average duration of mechanical ventilation between both strategies.

## Conclusion

There is significant variability in the practice of obtaining CXR in ICU patients in the Netherlands. The utility of daily-routine CXR ordering needs to be studied in a large, prospective fashion. Results from these studies will then inform appropriate guideline recommendations.

## Competing interests

The author(s) declare that they have no competing interests.

## Authors' contributions

MG participated in collection, analysis and interpretation of the data and in drafting the manuscript. KH contributed to conception and design of the study and revising of the manuscript. JK was involved in design and statistical analysis of the study. PS and JS have been involved in drafting and revising the manuscript critically for important intellectual account. PS and MS conceived and coordinated the study and were involved in the interpretation of the data and revising of the manuscript. All authors read and approved the final manuscript.

## Pre-publication history

The pre-publication history for this paper can be accessed here:



## Supplementary Material

Additional File 1 Survey (translated from Dutch language) Click here for file

## Figures and Tables

**Table 1:** Daily-routine versus on demand chest radiograph strategy according to type of intensive care unit and number of beds

	**Daily-routine strategy**	**On demand strategy**
all hospitals – N (%)	26 (63%)	15 (37%)
academic intensive care units – N (%)	5 (50%)	5 (50%)
*daily-routine strategy was practiced in*: all patients – N (%)	1	-
intubated and mechanically ventilated patients only – N (%)	1	-
distinctive patient groups only – N (%)	3	-
non-academic intensive care units – N (%)	21 (68%)	10 (32%)
*daily-routine strategy was practiced in*: all patients – N (%)	5	-
intubated and mechanically ventilated patients only – N (%)	14	-
distinctive patient groups only – N (%)	2	-
intensive care units 5 – 15 beds – N (%)	22 (65%)	12 (35%)
intensive care units 16 – 20 beds – N (%)	2 (66%)	1 (33%)
intensive care units > 20 beds – N (%)	2 (50%)	2 (50%)

daily-routine strategy; standing orders for obtaining a daily chest radiograph on demand strategy; all chest radiographs were made on clinical indications distinctive patient groups: this was not further specified in the survey

**Table 2:** Assumed value of daily-routine and on demand chest radiographs in academic and non-academic intensive care units

	**Daily-routine**		**On demand**	
	**Academic**	**Non-academic**	**Academic**	**Non-academic**

Assumed value*				
<10	17%	8%	0%	3%
10–20	66%	32%	60%	40%
21–30	17%	20%	10%	27%
30–60	0%	28%	20%	23%
>60	0%	12%	10%	7%

*, values are expressed as the percentage of chest radiographs that are assumed to lead to a change in therapy.
