# Supplementary material for: Chest radiography practice in critically ill patients: a postal survey in the Netherlands
Source: BMC Med Imaging. 2006 Jul 18;6:8. doi: 10.1186/1471-2342-6-8 (PMC1557847; doi:10.1186/1471-2342-6-8)
Supplement: Additional File 1 — Survey (translated from Dutch language) [file 1471-2342-6-8-S1.doc]

Additional file 1

Survey (translated from Dutch language):

Demographic questions

1. Number of ICU-beds available for mechanical ventilation: 🞎 < 5; 🞎 5 – 15; 🞎 15 – 20; 🞎 > 20
2. Type of hospital: 🞎 academic; 🞎 non-academic
3. Number of fellows in training for intensivist: 🞎 none; 🞎 1 – 4; 🞎 > 4
4. Type of ICU: 🞎 closed format; 🞎 open format

Questions regarding chest radiography

1. Regarding daily-routine chest radiography: 🞎 not performed in our ICU; 🞎 performed in our ICU for all admitted patients; 🞎 performed in our ICU, but only for intubated and mechanically ventilated patients; 🞎 performed in our ICU, but only in specific patient groups (specify).
2. A chest radiograph is always performed after: 🞎 endotracheal intubation; 🞎 insertion of a central venous line; 🞎 insertion of a pulmonary artery catheter; 🞎 insertion of a mini-tracheostomy; 🞎 insertion of tracheostomy; 🞎 insertion of chest tube; 🞎 insertion of other invasive devices; 🞎 ventilatory deterioration; 🞎 circulatory deterioration; 🞎 cardiopulmonary resuscitation; 🞎 arrival in ICU; 🞎 before removal of endotracheal tube; 🞎 after removal of endotracheal tube; 🞎 before ICU-discharge.
3. The daily routine chest radiograph influences care of patients in: 🞎 < 10%; 🞎 10 – 20%; 🞎 20 – 30%; 🞎 30 – 60%; 🞎 > 60%.
4. The on demand chest radiograph influences care of patients in: 🞎 < 10%; 🞎 10 – 20%; 🞎 20 – 30%; 🞎 30 – 60%; 🞎 > 60%.
5. A chest radiograph is essential for the judgment of: 🞎 presence or absence of ARDS; 🞎 presence or absence of pneumonia; 🞎 presence or absence of pneumothorax; 🞎 filling state of patient; 🞎 position of central venous lines; 🞎 position of chest tube; 🞎 position of intra-aortic counter pulsing device.
